# Salvianolic acid B and Senkyunolide I synergistically alleviate cardiac hypertrophy and regulate MAP3K1 signaling

**DOI:** 10.1186/s13020-025-01189-9

**Published:** 2025-09-28

**Authors:** Changtong Liu, Rui Guo, Yue Zhou, Miao Zhu, Li Shao, Yingchao Wang, Lu Zhao

**Affiliations:** 1 Pharmaceutical Informatics Institute, College of Pharmaceutical Sciences, Zhejiang University, Hangzhou, 310058 China; 2 Innovation Institute for Artificial Intelligence in Medicine of Zhejiang University, Hangzhou, 310018 China; 3 State Key Laboratory of Chinese Medicine Modernization, College of Pharmaceutical Sciences, Zhejiang University, Hangzhou, 310058 China; 4 School of Clinical Medicine, Hangzhou Normal University, The Affiliated Hospital of Hangzhou Normal University, Hangzhou, 311121 Zhejiang China; 5 Department of Pharmacy, Affiliated Hangzhou First People’s Hospital Chengbei Campus, School of Medicine, Westlake University, Hangzhou, 310000 Zhejiang China; 6 Department of Vascular Surgery, The Second Affiliated Hospital of Zhejiang University Medical School, Hangzhou, 310003 China; 7 Zhejiang Key Laboratory of Molecular Cancer Biology, Life Sciences Institute, Zhejiang University, Hangzhou, 310058 China

**Keywords:** Salvianolic acid B, Senkyunolide I, Cardiac hypertrophy, Zebrafish, Guanxinning tablet, Multi-model analysis

## Abstract

**Background:**

Cardiac hypertrophy, characterized by the thickening of the heart muscle, arises from factors such as hypertension and genetic mutations, often leading to adverse outcomes like heart failure and arrhythmias. Guanxinning tablets (GXNT), a botanical drug composed of the blood-activating herbs *Salvia miltiorrhiza* Bunge. and *Ligusticum striatum* DC., are widely used in the treatment of cardiovascular diseases. However, the active ingredients and their molecular mechanisms are yet to be fully understood.

**Methods:**

We evaluated the anti-hypertrophic effects of GXNT and screened its active substances via cardiac function live-imaging on an aristolochic acid A-stimulated zebrafish cardiac hypertrophy model, and through F-actin immunostaining on a phenylephrine-induced hypertrophic NRCMs model. Additionally, the protective effects of GXNT’s active substances were analyzed in a mouse model of cardiac hypertrophy using echocardiography, histopathology analysis, and western blotting.

**Results:**

The anti-hypertrophic effects of GXNT were assessed using an aristolochic acid A-stimulated zebrafish model and phenylephrine-induced hypertrophic NRCMs. GXNT demonstrated significant anti-hypertrophic effects in both models. Phenotypic screening identified Senkyunolide I (Sen I) from *Ligusticum striatum* as the active component in the zebrafish model, while Salvianolic acid B (Sal B) and Rosmarinic acid from *Salvia miltiorrhiza* emerged as the key anti-hypertrophic compound in NRCMs. In a mouse model of isoproterenol-induced cardiac hypertrophy, Sal B and Sen I showed synergistic effects, improving cardiac function, reducing oxidative stress, and suppressing inflammation. Mechanistically, transcriptomic sequencing highlighted cooperative modulation of MAP3K1 signaling by the two compounds. Notably, siRNA-mediated knockdown of MAP3K1 in cardiomyocytes attenuated the hypertrophic phenotype, supporting its essential role in the pathological process. Molecular docking and dynamic simulations further supported their binding potential to MAP3K1.

**Conclusion:**

These findings underscore GXNT’s potent anti-hypertrophic effects, possibly driven by the synergistic actions of Sal B and Sen I, and offer insights into its therapeutic potential through MAP3K1 signaling regulation.

**Supplementary Information:**

The online version contains supplementary material available at 10.1186/s13020-025-01189-9.

## Introduction

Cardiac hypertrophy remains a critical therapeutic challenge in cardiovascular diseases, representing a major risk factor for heart failure despite current pharmacological interventions [1, 2]. While conventional drugs like angiotensin-converting enzyme (ACE) inhibitors and β-blockers are effective at alleviating clinical symptoms, their inability to significantly reduce mortality rates underscores the need for novel therapeutic strategies targeting the complex pathophysiology of hypertrophy [3, 4]. This complexity arises from intertwined pathological mechanisms, including neuroendocrine activation, metabolic dysregulation, chronic inflammation, and aberrant kinase signaling [3, 5, 6].

The mitogen-activated protein kinase (MAPK) signaling network is critical in pathological hypertrophy by integrating diverse hypertrophic stimuli into coordinated transcriptional response. The MAPK cascade stimulates nuclear translocation of effector proteins like nuclear factor of activated T cells (NFAT) and activator protein-1, driving expression of pathological hypertrophy genes [5]. Within this network, mitogen-activated protein kinase kinase kinase 1 (MAP3K1) emerges as a pivotal regulator of pathological hypertrophy, with both pro-hypertrophic and cardioprotective functions reported. While MAP3K1 deficiency abolishes hypertrophy and preserves ventricular function in a G protein Gαq-dependent manner [7] and its inhibition by netrin-1 attenuates pathological remodeling [8], other work suggests protective functions through Cdc42–MAP3K1–JNK signaling that antagonizes calcineurin-NFAT [9]. Notably, MAP3K1 knockout mice develop comparable hypertrophy but worse heart failure after pressure overload [10], implying its primary protective role may also involve preventing maladaptive remodeling. These discrepancies likely reflect differences in experimental models and MAP3K1’s complex interaction within multiple signaling pathways.

Guanxinning tablets (GXNT) is a botanical drug (Chinese patent drug no. Z20150028) composed of extracts from *Salvia miltiorrhiza* Bunge (*Smil*, Dan-Shen) and *Ligusticum chuanxiong* DC (*Lstr*, Chuan-Xiong) and is clinically used for the treatment of coronary heart disease. Compared to the placebo group, the GXNT group showed significantly extended exercise duration and improved ECG manifestations in a multicenter randomized controlled trial (RCT) of angina pectoris patients [11]. Meanwhile, a pilot RCT demonstrated that GXNT significantly reduced the risk of residual inflammation among patients with atherosclerotic cardiovascular disease [12]. Notably, *Smil* and *Lstr* were widely-used herbal plants for promoting blood circulation and resolving stasis (termed “Huo-Xue Hua-Yu” in Chinese) in China and many other Asian countries for centuries [13]. Emerging evidence indicates that *Smil* mitigates cardiomyocyte ferroptosis and attenuates post-myocardial infarction injury by activating the Nuclear factor erythroid 2-related factor 2 pathway [14]. Its clinical formulation, Dan-shen Injection, effectively prevents heart failure following acute myocardial infarction [15]. In parallel, bioactive constituents from *Lstr*, especially liguzinediol [16] and lactones [17], demonstrate significant efficacy in alleviating myocardial injury. However, the widespread application of GXNT is hindered by two fundamental gaps: (1) unclear identification of active components, and (2) insufficient mechanistic understanding of how these components might interact therapeutically.

In this study, we propose a multi-model based approach to evaluate the anti-hypertrophic effects of GXNT and to screen for its active components. Using fluorescence immunostaining, probes, and fluorescein-labeled transgenic organisms, we examined the regulatory effects of GXNT and its components on cardiomyocytes hypertrophy in primary cultures of neonatal rat cardiomyocytes (NRCMs) and zebrafish. In recent years, the vertebrate model zebrafish has been increasingly used in cardiovascular researches due to its high similarity in the anatomical structure and biological function of cardiovascular system with those of mammals, as well as its unique advantages in drug screening and live imaging [18]. Through this approach, we identified salvianolic acid B (Sal B) and Senkyunolide I (Sen I) as the major active compounds mediating the anti-hypertrophic effects of GXNT. The endogenous effects and potential synergistic interaction of Sal B and Sen I were further investigated in a hypertrophic mice model. Overall, this study delves into the pharmacological substances in GXNT, as well as the interaction between its active compounds in the regulation of cardiac hypertrophy, aiming to elucidate the mechanisms and compatibility principles underlying a classical TCM formula.

## Materials and methods

### Mice

Male C57BL/6J mice were procured from Shanghai Slac Laboratory Animal Technology, China. The animals were maintained under standard laboratory conditions, like a 12-h light/dark cycle at 25 °C, with unrestricted access to food and water throughout the experiment.

### Zebrafish husbandry

*Tg*(*cmlc2: mCherry*) [19] was obtained from the Laboratory Animal Center of Zhejiang University. Zebrafish were kept and maintained according to standard protocols [20]. Zebrafish embryos were cultured in E3 medium (0.29 g/L NaCl, 0.013 g/L KCl, 0.048 g/L CaCl_2_·2H_2_O, 0.082 g/L MgCl_2_·6H_2_O, pH 7.2).

### Chemicals and reagents

Isoproterenol (ISO, 1351005), 1-phenyl 2-thiourea (PTU, P7629) and Ethyl 3-aminobenzoate methanesulfonate (Tricaine, E10521) were purchased from Sigma-Aldrich company of United States. Phenylephrine (PE, P106007) and Captopril (C8856) were purchased from Aladdin of China. Aristolochic acid A (AA, B20817), Danshensu (B20254), Sal B (B20261), Rosmarinic acid (B20862), Sen I (B21463) and Ferulic acid (B20007) were purchased from Shanghai Yuanye Biotechnology Co., Ltd. (Shanghai, China). The purities of these six compounds are greater than 98%. Anti-GAPDH (AF0006) antibody and MDA (S0131) were purchased from Beyotime Company of Shanghai, China. Digoxin (D1828) was purchased from Tokyo Chemical Industry Co., Ltd of Japan. DCFH-DA (MA0219) was purchased from Dalian Meilun Biotechnology Company, China. Alexa Four 488 Phalloidin (8878S) was purchased from Cell Signal Technology of United States. MAP3K1 Polyclonal antibody (19970-1-AP) and MAX Polyclonal antibody (10426-1-AP) were purchased from Proteintech of United States.

### Preparation of GXNT and the extracts of Smil and Lstr

GXNT and herb extracts were produced by Chiatai Qingchunbao Pharmaceutical Co. Ltd. (Hangzhou, China, batch number 23003), following the guidelines of the National Medical Products Administration of China (YBZ00342016). The rhizomes of *Smil, Lstr,* or the two herbal compositions at 1:1 weight ratio (for GXNT), were collected and extracted with water for three times. The extracts of the three rounds were mixed, filtered, and concentrated at 50 °C to reach a relative density between 1.20 and 1.25. The condensed extract was further precipitated with ethanol, and the supernatant was concentrated again at 50 °C to reach a relative density between 1.25 and 1.35. Finally, a vacuum drying oven was used to dry the concentrated extracts to powder. Each gram of GXNT is derived from about 6.46 g of herbs (3.23 g *Smil* and 3.23 g *Lstr*). Each gram of *Smil* extract is derived from about 10.36 g of *Smil* raw material and each gram of *Lstr* extract is derived from about 6.97 g of *Lstr* raw material.

### Zebrafish cardiac hypertrophy modeling

Embryos were obtained through natural spawning. Zebrafish larvae of 54 h post-fertilization (hpf) were arrayed into 12-well plates (8 fish per well) and were treated with 50 μM AA, and 0.1%DMSO was served as a vehicle control. At 72 hpf, fluorescent heart-beating images were acquired by Leica DMI 3000B inversed microscope system (Leica Microsystems, Germany). Zebrafish larvae at 54 hpf were randomly divided into the following groups: control, AA-induced hypertrophy model, treatment group. All compounds were dissolved in E3 medium and administered by immersion for 18 h. The GXNT dose of 1000 μg/mL was selected based on a conversion formula reported previously [21]. Experimental validation confirmed that 1000 μg/mL was both effective and safe. Doses for Sal B and Sen I (100 μg/mL) were chosen based on prior studies demonstrating efficacy in cardiovascular models [22–24] and confirmed to be non-toxic in preliminary tests.

### Analysis of zebrafish cardiac function parameters

Zebrafish larvae were temporarily anesthetized with tricaine (300 μM; A5040, Sigma-Aldrich, USA) during imaging to ensure stillness. Cardiac function parameters were assessed over at least four consecutive cardiac cycles, encompassing both systole and diastole. The evaluation was conducted following the method described previously [25, 26]. In brief, end-systole (ES) and end-diastole (ED) frames were manually identified as the frames corresponding to the last forward flow from the outflow tract and the last inflow into the ventricle from the atrium, respectively. The ventricles area in ES (ESA) and ED (EDA) frames was segmented based on the endocardium boundary highlighted by cardiomyocytes fluorescence. Pixel counts were used to measure the ventricular area. A rotated minimum-area rectangle was fitted around the segmented ventricle to calculate the long axis (a) and short axis (b). Heart rate (beats per minute, BPM) was determined by counting the number of contractions over a 2s heart-beating video.

$$ {\text{Ventricular\;fractional\;area\;change}},\;{\text{FAC}} = \frac{{{\text{EDA}} - {\text{ESA}}}}{{{\text{EDA}}}} \times {1}00\% $$

$$ {\text{Fractional\;shortening}},{\text{FS}} = \frac{{{\text{EDa}} - {\text{ESa}}}}{{{\text{EDa}}}} \times {1}00\% $$

$$ {\text{ED.Vol}} = \frac{{4{\uppi }}}{3}\left( {{\text{EDa}} \times {\text{EDb}}^{2} } \right);\;{\text{ES}}{\text{.Vol}} = \frac{{4{\uppi }}}{3}\left( {{\text{ESa}} \times {\text{ESb}}^{2} } \right) $$

$$ {\text{Stroke\;volume}},\;{\text{SV}} = {\text{ED}}{\text{.Vol}} - {\text{ES}}{\text{.Vol}} = \frac{{4{\uppi }}}{3}\left( {{\text{EDa}} \times {\text{EDb}}^{2} - {\text{ESa}} \times {\text{ESb}}^{2} } \right) $$

$$ {\text{Cardiac\;output}},\;{\text{CO}} = {\text{SV}} \times {\text{BPM}} $$

### Survival of zebrafish

Starting 18 h after AA treatment, the survival status of zebrafish embryos in each group was recorded every 30 min until 32 h. Survival curves were plotted using GraphPad Prism 8 software, and the survival data between the model group and each treatment group were statistically analyzed using the Log-rank (Mantel-Cox) test.

### Paraffin embedding and histological staining of zebrafish

Zebrafish embryos at 72 hpf, 18 h post-AA treatment, were fixed overnight in 4% paraformaldehyde. After fixation, the embryos were subjected to a standardized dehydration and decolorization process. The specimens were then vertically embedded, head-down, in melted paraffin and allowed to solidify. Once the paraffin was fully solidified, the embedded tissues were sectioned, stained with hematoxylin and eosin, and mounted for examination. Histological analysis and imaging were performed under a microscope at 20X magnification.

### Cell culture and treatment

NRCMs were isolated from the hearts of neonatal rats (1 to 3 days, Sparague–Dawley rats, either sex) using the Neonatal Heart Dissociation Kit (MACS, Germany), following the manufacturer’s protocol. The cardiomyocytes were plated in gelatin-coated plates in a plating medium containing 10% FBS. After pre-plating for 30 min to remove fibroblasts, unattached cardiomyocytes were collected and plated onto fibronectin-coated culture plates. Cardiomyocyte cultures were used after 24 h of plating. For PE treatment, NRCMs were seeded into 96-well plates at a density of 5000 cells per well, and PE (100 μM) was added for 48 h to induce the cardiac hypertrophy. DMSO (0.1%) was added as the blank control. For rescue experiments in vitro, compounds were used at the doses indicated in each figure.

### Measurement of cell surface area by immunofluorescence

After 48 h of PE treatment, the cultured NRCMs were fixed with 4% paraformaldehyde for 30 min at room temperature, followed by permeabilization with 0.1% Triton X-100 for 5 min. Cells were then stained with Alexa Fluor 488 Phalloidin (1:20) overnight at 4 °C. Then, the cells were washed and stained with Hoechst (1:10,000) at 37 °C for 10 min. Fluorescent images were captured by ImageXpress Pico Automated Cell Imaging System (Molecular Devices, USA), and the surface areas were measured with the imaging system supporting software CellReporterXpress®.

### Quantitative real-time PCR analysis

Total RNA was extracted from NRCMs using an RNA-Quick Purification Kit (RN001, ES Science, China) following the manufacturer’s instructions. The total RNA concentration was measured using a NanoDrop 2000 spectrophotometer, and subsequently reverse-transcribed into single-strand cDNA with HiFiScript cDNA Synthesis Kit (CW2569M, CWBIO, China). Real-time PCR was conducted using the two-step quantitative RT-PCR method with 2 X SYBR Green qPCR Mater Mix (B21202, Bimake, USA). GAPDH served as the internal control for normalization. The cycling protocol need to hatch at 95 °C for 5 min followed by 40 cycles of 95 °C for 10 s and 60 °C for 30 s. Relative mRNA expression levels of target gene were normalized to GAPDH. Primer sequences are as follows: GAPDH: 5ʹ-TCCACCACCCTGTTGCTGTAGC-3ʹ; 5ʹ-TGGAAAGCTGTGGCGTGATG-3ʹ; ANF: 5ʹ-GGGAAGTCAACCCGTCTCA-3ʹ; 5ʹ-GGCTCCAATCCTGTCAATCC-3ʹ; BNP: 5ʹ-AAGCTGCTGGAGCTGATAAGA-3ʹ; 5ʹ-GTTACAGCCCAAACGACTGAC-3ʹ; MAP3K1: 5ʹ-TACACTCCTTGCCACAGTCTGG-3ʹ; 5ʹ-CCTTGCAGAGTTCCAGCACTGT-3ʹ; MAPK11: 5ʹ-AGCAATGTAGCGGTGAACGA-3ʹ; 5ʹ-TCAGCTGGTCGATGTAGTCG-3ʹ; MAX: 5ʹ-ACATCGAGGTGGAGAGCGA-3ʹ; 5ʹ-CTCCAGTGCACGGACTTGTT-3ʹ.

### Measurements of intracellular ROS

NRCMs were plated in 96-well plates and treated with the above-mentioned method. ROS level was detected by 2ʹ,7ʹ-dichlorodihydrofluorescein diacetate (DCFH-DA, MeilunBio, China) according to manufacturer’s instructions. Fluorescent images were acquired by ImageXpress Pico Automated Cell Imaging System (Molecular Devices, USA), and the surface areas were measured with the imaging system supporting software CellReporterXpress®.

### MDA analysis

Discard the cell supernatant and wash the cells three times with PBS. Cells were lysed in the Cell Lysis Buffer for Western and Immunoprecipitation (IP) (P0013, Beyotime, China). Collect the cell lysate, then centrifuge at 12,000 rpm, 4 °C, for 10 min. Subsequently, the level of MDA was analyzed by the MDA Detection Kit (S0131, Beyotime, China), and measured by Tecan Infinite M1000 PRO microplate reader.

### ISO-induced cardiac hypertrophy mouse model

The cardiac hypertrophy model was established using a gradient induction method as reported before [27], involving subcutaneous injections of ISO at a dose of 20 mg/kg on the first day, 10 mg/kg on the second day, followed by daily injections of ISO at 5 mg/kg for 3 weeks. Male C57BL/6 mice were randomly divided into six groups based on body weight, with 12 mice in each group: Control group, ISO group (Model), ISO + Sal B group, ISO + Sen I group, ISO + Sal B + Sen I (combination) group, and ISO + captopril (positive control) group. The Control group received equivalent doses of saline subcutaneously. Sal B was administered at a concentration of 80 mg/kg/day, Sen I at a concentration of 50 mg/kg/day, and captopril at a concentration of 20 mg/kg/day. The doses of Sal B (80 mg/kg) and Sen I (50 mg/kg) were selected based on prior studies demonstrating their effective cardioprotective effects in murine models [22–24]. The Control and Model groups received intraperitoneal injections of saline as controls. Mice were weighed daily before administration, and the injection volume was adjusted according to the daily body weight. Echocardiography was performed using a Vevo 2100 system (VisualSonics, USA) at the end of the treatment.

### Drug interaction analysis

Assuming that the control group demonstrates a 100% therapeutic effect compared to the ISO group (model), all other groups were normalized to the control group as a percentage. For each group, the rescue effects E was calculated by (value of treatment group)/(value of control group) * 100%. The drug interaction is calculated based on the Bliss Independence model [28]. The observed drug combination effect is expressed as E_Sal·Sen_(0 ≤ E_Sal·Sen_ ≤ 1). In this model, the threshold for the predicted combined effect (without synergism) of the two drugs is defined as E_Sal_ + E_Sen_(1 − E_Sal_) = E_Sal_ + E_Sen_ − E_Sal_E_Sen_, where (0 ≤ E_Sal_ ≤ 1, 0 ≤ E_Sen_ ≤ 1). The combination index (CI) is calculated as (E_Sal_ + E_Sen_ − E_Sal_ × E_Sen_)/E_Sal·Sen_. The dotted line represents the value of E_Sal_ + E_Sen_ − E_Sal_ × E_Sen_. t-test was performed to compare the observed effect with the additive effect, and the p-value was shown on corresponding Figs. A CI value of less than 1 indicates that the two compounds exhibit some degree of synergy. When the combined effect, E_Sal·Sen_, exceeds this threshold and is statistically significant, it suggests that the combination therapy is superior to either monotherapy alone.

### Histopathology analysis

For histological analysis, hearts were fixed in 4% paraformaldehyde (pH 7.4) overnight, embedded in paraffin, and cut into serial sections of 5 μm thickness. Hematoxylin and Eosin (H&E) taining was performed to observe morphological changes. The changes in collagen fibers in cardiac tissue was assayed by picric acid-picrosirius red staining. Immunohistochemistry images were captured with SLIDEVIEW VS200 (Olympus, Japan) and analyzed by a person blinded to treatment using Image J.

### Western blotting

Myocardial tissue (approximately 20 mg) was lysed in cold RIPA lysis buffer supplemented with1% phenylmethylsulfonyl fluoride (PMSF; ST505, Beyotime, China) and 1% protease inhibitor cocktail (HY-K0010, MedChemExpress, USA) to extract the total protein. Equal amounts of protein were loaded onto SDS-PAGE gels, separated, and transferred to polyvinylidene difluoride (PVDF) membranes. The membranes were then blocked and incubated overnight at 4 °C with primary antibodies, followed by incubation with secondary antibodies for 1 h at room temperature. Enhanced chemiluminescence (ECL) reagents were used for signal detection, and the blot intensity was quantified using a ChemiDoc MP Imaging System (Bio-Rad, USA).

### Transcriptomic sequencing and data analysis

RNA-seq-based transcriptome analysis of heart tissues were collected after ISO treatment. Total RNA was isolated using TRIzol reagent (CW0580S, CWBio, China). RNA degradation and contamination was monitored on 1% agarose gels. RNA purity was checked using the NanoPhotometer® spectrophotometer (IMPLEN, CA, USA). A total amount of 1 µg RNA per sample was used as input material for the RNA sample preparations. Sequencing libraries were generated using NEBNext® UltraTM RNA Library Prep Kit for Illumina® (NEB, USA) following manufacturer’s recommendations and index codes were added to attribute sequences to each sample. Transcriptome sequencing and analysis were conducted by OE Biotech Co., Ltd. (Shanghai, China).

Differentially expressed genes (DEGs) analysis was performed using the DESeq2 R package (1.16.1). DESeq2 provide statistical routines for determining differential expression in digital gene expression data using a model based on the negative binomial distribution. The resulting P-values were adjusted using the Benjamini and Hochberg’s approach for controlling the false discovery rate. Genes with an adjusted P-value < 0.05 found by DESeq2 were assigned as differentially expressed. We used clusterProfiler R package to test the statistical enrichment of differential expression genes in KEGG pathways.

### siRNA-mediated knockdown experiments

Small interfering RNAs (siRNAs) targeting rat MAP3K1 were designed and synthesized (Sangon Biotech, Shanghai, China). Three distinct siRNA sequences (siMAP3K1-#1, siMAP3K1-#2, and siMAP3K1-#3) were employed to ensure specificity and efficiency. A non-targeting scrambled siRNA (si-NC) was used as a negative control. SiRNAs sequences are as follows: siMAP3K1-#1 s, 5ʹ-GGCGUAGCUCAAGAAUCAAtt-3ʹ; siMAP3K1-#1a, 5ʹ-UUGAUUCUUGAGCUACGCCtt-3ʹ; siMAP3K1-#2 s, 5ʹ-GGUCGAGAGAUGGAGAAUAtt-3ʹ; siMAP3K1-#2a, 5ʹ-UAUUCUCCAUCUCUCGACCtt-3ʹ; siMAP3K1-#3s, 5ʹ-GAGACAGUCCAGACAAUAAtt-3ʹ; siMAP3K1-#3s, 5ʹ-UUAUUGUCUGGACUGUCUCtt-3ʹ; si-NCs, 5ʹ-UUCUCCGAACGUGUCACGUtt-3ʹ; si-NCa, 5ʹ-ACGUGACACGUUCGGAGAAtt-3ʹ. NRCMs were isolated and seeded in 6 or 96-well plates and transfected at approximately 60–70% confluency using Lipofectamine™ RNAiMAX Transfection Reagent (13778150, Invitrogen, USA) according to the manufacturer’s instructions. Briefly, siRNAs (final concentration: 25 nM) were diluted in Opti-MEM™ (31985062, Gibco, USA) and incubated with Lipofectamine™ RNAiMAX for 5 min at room temperature before adding to the cells. After 48 h of incubation, the medium was replaced with fresh complete culture medium, and cells were further incubated for 48 h. Knockdown efficiency was assessed by western blotting.

### Molecular docking assay

Molecular docking was performed by AutoDock Vina 1.2.5, which supports the simultaneous docking of multiple ligands [29]. The three-dimensional (3D) structure of MAP3K1 (PDB code: 6WHB) was retrieved from the RCSB PDB database (https://www.rcsb.org/), Moreover, the 2D structure of Sal B and 3D structure of Sen I (PubChem CID: 6451084 and 11521428, respectively) were collected from the PubChem database (https://pubchem.ncbi.nlm.nih.gov/), and the 3D structure of Sal B was generated by Chem3D Ultra 2022 (PerkinElmer Inc.).For initial preparation, PyMOL 3.0.3 and AutoDockTools 1.5.7 [30] were employed to prepare the retrieved proteins and compounds, which including hydrogen addition, charge calculation and solvent molecule deletion. The potential binding pockets were predicted by the DoGSiteScorer online tool (https://proteins.plus/) and pocket with the best score was applied to establish the grid box. In addition, to meet the supported formats of preparation software, the retrieved compounds were converted to MOL2 files using Open Babel 3.1.0 [31]. Ultimately, to acquire more accurate docking results, the maximum number of binding modes to generate was set to 50, with a maximum allowable energy range of 3 kcal/mol. The visualization of docking results was realized using PyMOL 3.0.3 and Discovery Studio 2021 Client (Neotrident Technology Ltd.).

### Molecular dynamic simulation

Molecular dynamics (MD) simulation for Sal B and Sen I with MAP3K1 was conducted using Gromacs 2022 [32]. Loop regions of MAP3K1 were modeled using MODELLER 10.5, which involved optimizing the loop conformation within the context of the overall protein structure [33]. Moreover, the topology file of MAP3K1 was generated with the CHARMM36 force field (charmm36-jul2022.ff) [34] through GROMACS, and topology files of the two ligands were generated by Sobtop 1.0 (http://sobereva.com/soft/Sobtop/). The protein–ligand complex was consisted of MAP3K1 with the two ligands, and was solvated in a dodecahedron box with SPC/E water molecules and a 10 Å margin. Additionally, sodium and chloride ions were added to the complex with a concentration of 0.145 M to neutralize the charge of the system. Then the steepest descent algorithm was used to minimize energy of the solvated system with a maximum number of 50,000 steps. Next, the system was equilibrated through 100-ps NVT and 100-ps NPT ensemble simulation. Ultimately, the production MD simulation of the complex was performed for 100 ns with a time-step of 2 fs, ensuring the system had sufficient time to reach a statistically significant state. To explore the dynamical behavior of the complex, the root-mean-square deviation (RMSD) and the root-mean-square fluctuation (RMSF) values were calculated separately for the protein combined with each ligand after performing least squares fit to the backbone atoms.

### Statistical analysis

All data were presented as means ± standard error of the mean (SEM). Statistical analyses were conducted using one-way analysis of variance (ANOVA) with GraphPad Prism 8.0.2 (USA). A p-value of < 0.05 was regarded as statistically significant.

## Results

### GXNT and Sen I attenuate aristolochic acid A-stimulated cardiac hypertrophy zebrafish model

**Fig. 1:**
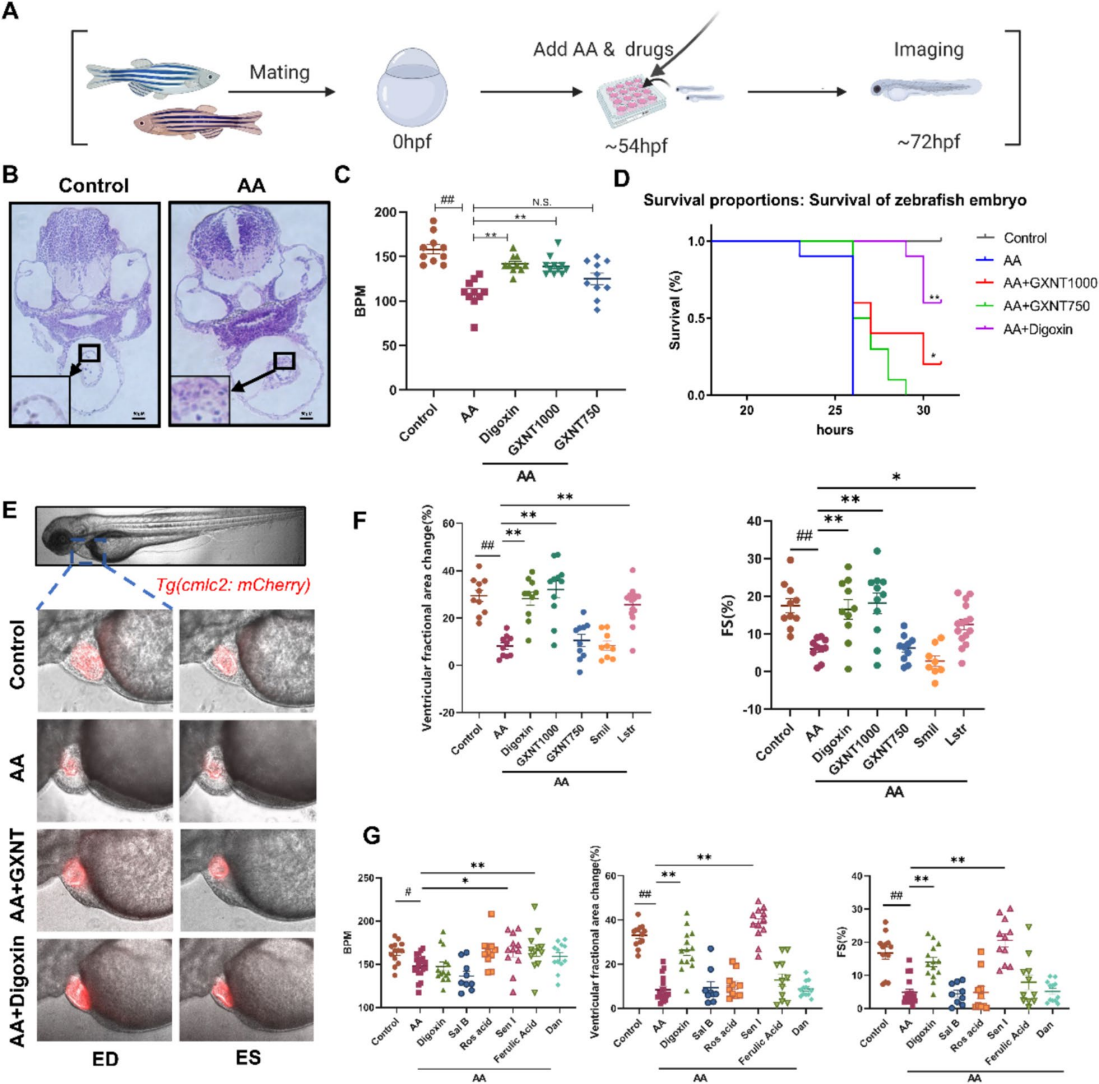
Identification of the Heart Protective Effects of GXNT and Sen I in AA-treated Zebrafish. **A** Schematic diagram of the experiment. **B** Representative hematoxylin and eosin (H&E)-stained images of zebrafish cardiac tissue sections. A magnified view of a section of the heart wall is shown in the lower left corner. AA, 50 μM. Scale bar, 100 μm. **C** Heart rate measurement of zebrafish embryos (n = 10 per group). BPM, beat per minute; AA, 50 μM; Digoxin, 10 μM; GXNT, 750 or 1000 μg/mL. **D** Kaplan–Meier survival curves of zebrafish embryos of different treatment. Digoxin, 10 μM; GXNT, 750 or 100 μg/mL; AA, 10 μM. n = 10 per group. **E** Representative fluorescence microscopy images of *Tg(cmlc2: mCherry)* zebrafish embryos. ED, end diastole; ES, end systole. **F** Effects of GXNT, *Smil*, and *Lstr* extracts on the zebrafish cardiac hypertrophy model (n = 10 per group). AA, 50 μM; Digoxin, 10 μM; GXNT, 750 or 1000 μg/mL;The concentrations of *Smil* and *Lstr* were 312 μg/mL and 463 μg/mL respectively. FS, fractional shortening. **G** Effects of GXNT compounds on the zebrafish cardiac hypertrophy model (n = 11–14 per group). The concentration of all compounds is 100 μg/mL. Quantitative data are presented as the mean ± SEM. Statistical significance was analyzed using one-way ANOVA with Tukey’s post hoc test; ^#^P < 0.05, ^##^P < 0.01 when compared with the control group; *P < 0.05, **P < 0.01 when compared with the AA group; ns, non-significant

Zebrafish have been increasingly used in the study of cardiovascular diseases and treatments [18, 35], due to their small size, larval transparency, rapid development, and high conservation with mammals. Previous studies have reported that AA treatment can induce cardiac hypertrophy and gradual loss of contractility in zebrafish larvae, accompanied with increased expression of proinflammatory genes, which effects can be rescued by ACE inhibitors and β-adrenergic receptor antagonists [36], suggesting the potential value of this model in the study of cardiac hypertrophy treatment. We thus used AA to optimize the modeling strategy of a zebrafish cardiac hypertrophy model. Significant thickening of the zebrafish heart ventricle and an increase in cardiomyocyte area were observed in zebrafish larvae after administering 50 μM AA at 54 hpf for 18 h (Fig. 1A, B). Additionally, we observed decreased heart rate in AA-treated zebrafish larvae, and the survival rates significantly dropped after extended AA treatment for over 25 h (Fig. 1C, D). Using a conversion formula reported previously [21], we determined the testing concentration of GXNT on fish based on its clinical dosage, and digoxin was chosen as the positive drug. Notably, significant rescue effects were observed for both heart rates and survival rates after GXNT treatment (Fig. 1C, D).

To accurately assess the impact of AA on cardiac function, we took advantage of zebrafish live imaging and evaluated multiple heart function parameters in the *Tg*(*cmlc2: mCherry*) transgenic line with fluorescein-labeled cardiomyocytes. Based on heart beating videos, the ventricular fractional area change, fractional shortening (FS), stroke volume (SV), and cardiac output (CO) were analyzed [37]. Severe contractility loss was detected in AA-treated larvae, suggesting the development of heart failure. However, GXNT at 1000 μg/mL significantly improved all the above heart function parameters, demonstrating potent heart protective effects (Fig. 1E, F and Figure S1).

To illustrate the active components in GXNT, we continued to examine the anti-hypertrophic effects of *Smil* and *Lstr* extracts, the two herbs contained in GXNT. Interestingly, *Lstr*, but not *Smil*, showed significant protective effects on the cardiac function parameters of AA-treated zebrafish (Fig. 1F and Figure S1). The effects of the chemical compounds in GXNT were tested subsequently. As the chemical ingredients in GXNT has been analyzed by high-resolution mass spectrometry in our previous research [38], we selected five compounds of the major peaks, including danshensu (Dan), Sal B, rosmarinic acid (Ros acid), Sen I, and ferulic acid, as the candidates for further experiments. Among these compounds, Sal B, Ros acid, and Dan are derived from *Smil*, while Sen I and ferulic acid are derived from *Lstr* [11]. As a result, Sen I, a natural phthalide derived from *Lstr*, significantly alleviated abnormal heart rates and ventricular contraction defects caused by AA, helping to maintain normal cardiac output (Fig. 1G and Figure S2).

### GXNT, Sal B and Ros acid attenuate phenylephrine-induced cardiac hypertrophy in vitro

**Fig. 2:**
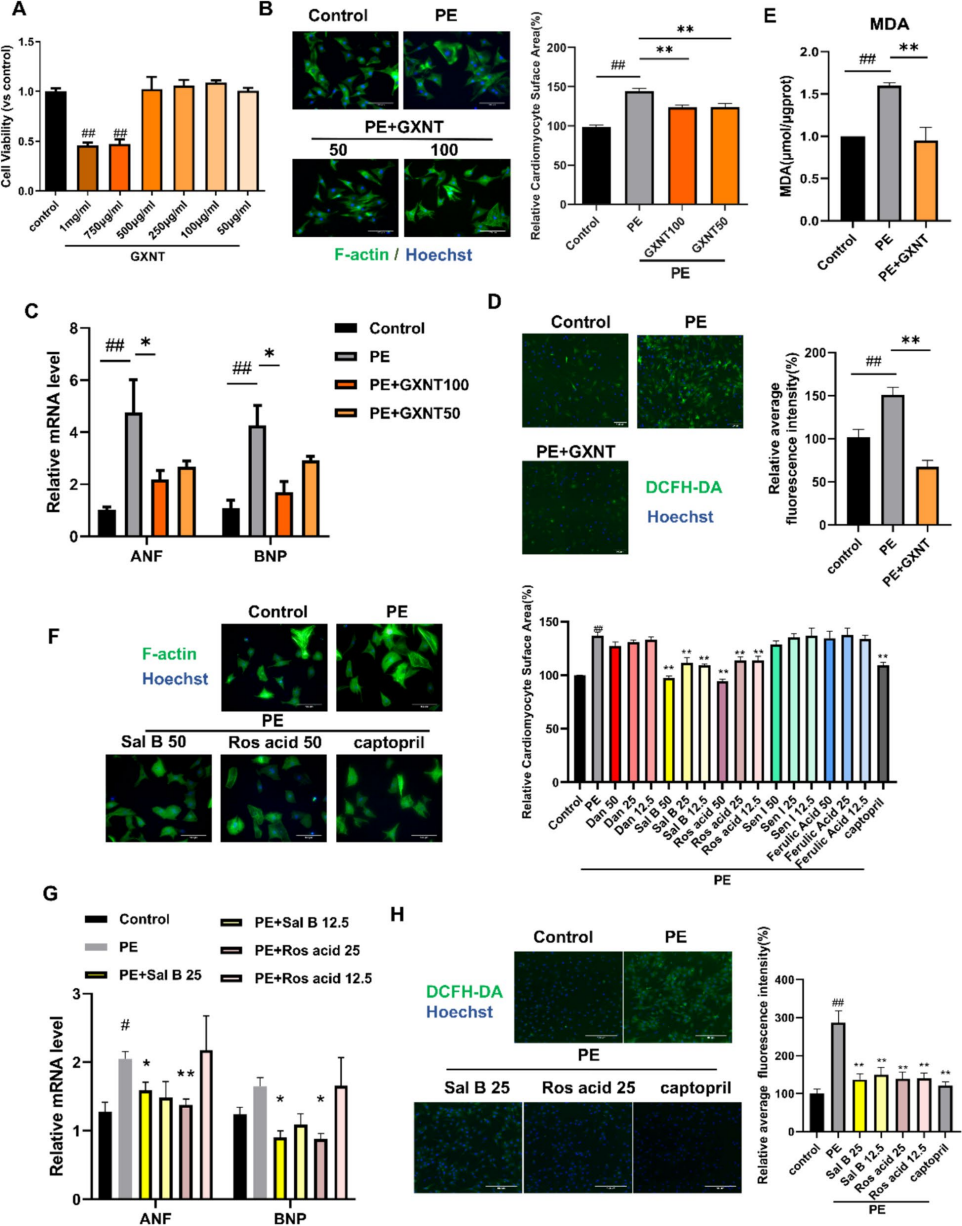
GXNT, Sal B and Ros acid attenuate phenylephrine-induced cardiac hypertrophy in NRCMs. **A** Toxicity analysis of GXNT in NRCMs. **B** Representative fluorescence images and corresponding quantification of phalloidin (F-actin) immunostaining in NRCMs. PE, 100 μM; GXNT,50 or 100 μg/mL. **C** Relative levels of ANF and BNP mRNA. **D** Representative images and corresponding quantification of DCFH-DA staining in NRCMs. GXNT, 100 μg/mL. **E** MDA concentration in NRCMs. GXNT, 100 μg/mL. **F** Representative phallodin immunostaining images and corresponding quantification of NRCMs treated with GXNT compounds. The concentrations of Sal B and Ros acid is 50 μM in the representative image, and captopril is used at 50 μM. **G** Relative levels of ANF and BNP mRNA in NRCMs. **H** Effects of Sal B and Ros acid on the level of ROS detected by DCFH-DA probe. The concentration of Sal B and Ros acid is 25 μM in the representative image, and captopril is used at 50 μM. Quantitative data are presented as the mean ± SEM. Statistical significance was analyzed using one-way ANOVA with Tukey’s post hoc test; ^##^P < 0.01 when compared with the control group; *P < 0.05, **P < 0.01 when compared with the PE group. Scale bar, 100 μm

Next, the potential effect of GXNT on cardiac hypertrophy was investigated at the cellular level in a PE-stimulated hypertrophic model in NRCMs. Toxicity analysis determined that 50 μg/mL and 100 μg/mL GXNT were safe concentrations for further testing (Fig. 2A). Immunostaining with the actin cytoskeleton antibody F-actin (Phalloidin) revealed that PE treatment increased cardiomyocyte size by over 40%, which was significantly reduced by GXNT treatment (Fig. 2B). Since the enlargement of cardiomyocyte size is typically accompanied by elevated expression of myocardial hypertrophy markers atrial natriuretic factor (ANF) and brain natriuretic peptide (BNP), the expression levels of the two factors were examined. PE treatment greatly induced the expression of both ANF and BNP, whereas GXNT significantly suppressed these changes (Fig. 2C). As the pathological development of cardiac hypertrophy is associated with elevated oxidative stress, we further examined the effects of GXNT on oxidative stress following PE treatment. Fluorescence analysis with the DCFH-DA probe demonstrated that GXNT markedly reduced PE-induced reactive oxygen species (ROS) accumulation in NRCMs (Fig. 2D), as well as the increase of lipid peroxidation (Fig. 2E). Collectively, these findings suggest that GXNT can attenuate PE-induced hypertrophy and oxidative stress in NRCMs.

We subsequently evaluated the effects of GXNT compounds on PE-induced hypertrophic NRCMs, with captopril used as the positive control. Salvianolic acid B (Sal B) and rosmarinic acid (Ros acid) significantly reduced cardiomyocyte size (Fig. 2F), downregulated ANF and BNP expression (Fig. 2G), and decreased ROS levels (Fig. 2H), suggesting that they exhibited potent anti-hypertrophic and anti-oxidative effects in NRCMs. Interestingly, these findings differ from those observed in the AA-induced hypertrophic zebrafish model. The varied effects of Sen I, Sal B, and Ros acid between NRCMs and zebrafish may be related to several factors, such as the differences in modeling approaches, mechanisms of drug activity, and/or the pathological processes between in vivo and in vitro models. In order to integrate the positive hits from different models, and examining the pharmacological basis of GXNT’s formulation, which consists of *Lstr* and *Smil*, we decided to include one active compound from each herb for further study. Among the three compounds, Sen I is derived from *Lstr*, whereas both Sal B and Ros acid are derived from *Smil*. As the quantity of Sal B in *Smil* is higher than that of Ros acid [39], Sal B is selected as a major active compound from *Smil*, and combined with Sen I from *Lstr* for the following validation and mechanistic studies in the mice model.

### Impact of Sal B and Sen I on cardiac hypertrophy in an isoproterenol treated mouse model

**Fig. 3:**
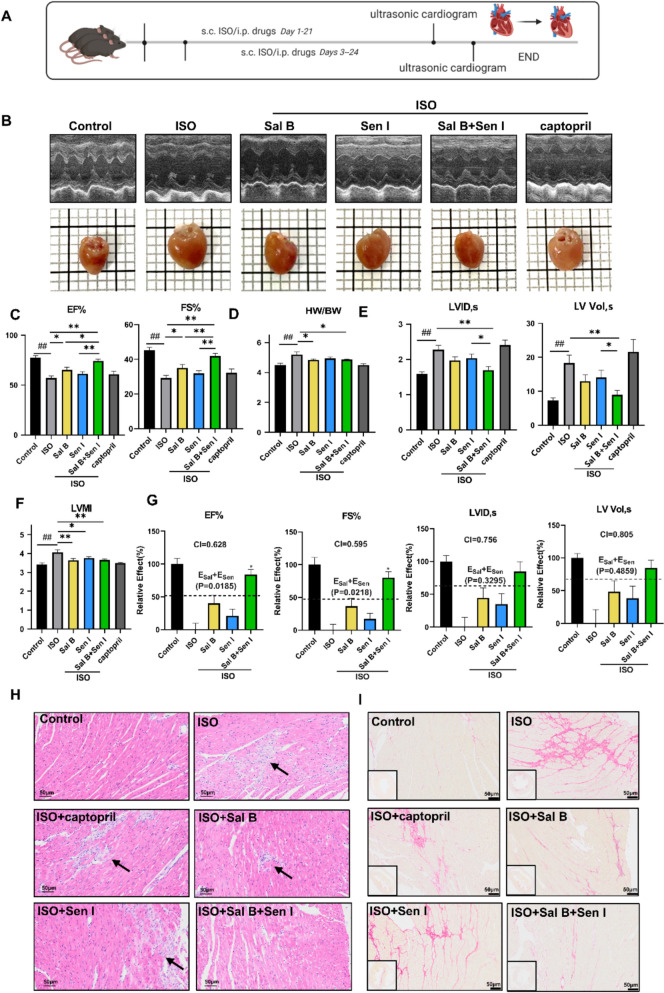
Impacts of Sal B and Sen I on cardiac hypertrophy in an ISO treated mouse model. **A** Schematic diagram of the experiment. **B** Representative images of echocardiography and heart tissues in mice. **C** The quantitative results of echocardiographic analyses (n = 9–12 per group). **D** The ratio of heart weight to body weight (HW/BW) in different groups of mice models. n = 9–12 per group. **E** The quantitative results of echocardiographic analyses (n = 9–12 per group). **F** Left ventricular mass index (LVMI) in different groups of mice models. n = 6–9 per group. **G** Analysis of the interaction between Sal B and Sen I in regulating heart parameters. n = 9–12 per group. **H** Representative H&E-stained images of mice cardiac tissue sections. Black arrowheads denote perivascular inflammatory cell infiltration. Scale bar, 50 μm. **I** Representative images of Sirius red staining of heart sections. Scale bar, 50 μm. An overall view of the heart cross-section is shown in the lower left cornerThe predicted additive effects for Sal B and Sen I (E_sal_ + E_sen_) is represented by a black dashed line. Sal B, 80 mg/kg/day; Sen I, 50 mg/kg/day; captopril, 20 mg/kg/day. Quantitative data are presented as the mean ± SEM. Statistical significance was analyzed using Student’s t-test in **G** and one-way ANOVA with Tukey’s post hoc test in **C**, **D**, **E** and **F**; ^##^P < 0.01 when compared with the control group; *P < 0.05, **P < 0.01

ISO, a β-receptor agonist, can induce cardiac hypertrophy with long-term low-dose administration and is currently used to establish stable models of cardiac hypertrophy [40]. Here we established an ISO-induced mouse cardiac hypertrophy model using a gradient modeling method, with subcutaneous injection of 20 mg/kg/day on the first day, 10 mg/kg/day on the second day, and then continuous injection of ISO (5 mg/kg/day) for 3 weeks (Fig. 3A). The heart weight was increased in the ISO-treated group, with reduced left ventricular contractile function, as indicated by decreased EF% and FS%. The doses of Sal B (80 mg/kg) and Sen I (50 mg/kg) were selected based on prior in vivo studies demonstrating their cardioprotective efficacy in murine models[22–24]. Treatment with Sal B, as well as the combination of Sal B and Sen I, significantly alleviated these alterations (Fig. 3B–D). Besides, ISO also increased the left ventricular internal diameter during systole (LVID, s), the left ventricular end-systolic volume (LV Vol, s), and left ventricular mass index (LVMI), and coadministration of Sal B and Sen I showed varied degrees of protection against the above changes (Fig. 3E, F). Interestingly, the combined usage of Sal B and Sen I provided significant increased protection of multiple parameters than single treatment. In order to further quantify whether the combined usage of Sal B and Sen I exhibits a synergistic effect, the synergy index (CI) was calculated using the Bliss Independence model [28]. As a result, synergistic effects (CI value < 1) was suggested for Sal B and Sen I for four cardiac parameters (EF%, FS%, LVID,s, LV Vol, s). The combined effect of Sal B and Sen I also significantly exceeds the predicted additive effect (E_sal_ + E_sen_) in EF% (p:0.0185) and FS% (p:0.0218) (Fig. 3G).

The pathological manifestation of heart tissue was further examined in ISO-stimulated mice model. Severe cardiac tissue damage, characterized by disorganized sarcomeres, was observed in ISO-treated mice. Treatment groups showed improved cardiac structure, with the most pronounced effect observed in the Sal B and Sen I combined group (Fig. 3H). Myocardial fibrosis was also examined by Sirius Red staining. In the control group, myocardial fibers were arranged neatly and densely, with no significant collagen fiber deposition. In contrast, the ISO group exhibited sparse myocardial cell arrangement, widened intercellular spaces, and marked proliferation of collagen fibers in the left ventricular muscle. In the treatment groups, particularly the Sal B and Sen I coadministration group, myocardial cell arrangement was largely rescued to the normal level, and the accumulation of ventricular collagen fiber signals was significantly reduced (Fig. 3I and Figure S3). Collectively, these findings suggest that Sal B and Sen I exhibited varying degrees of protective effects in the mice hypertrophic model, with the combination showing significant better results than the individual treatments.

### Coadministration of Sal B and Sen I regulates the MAP3K1 signaling

**Fig. 4:**
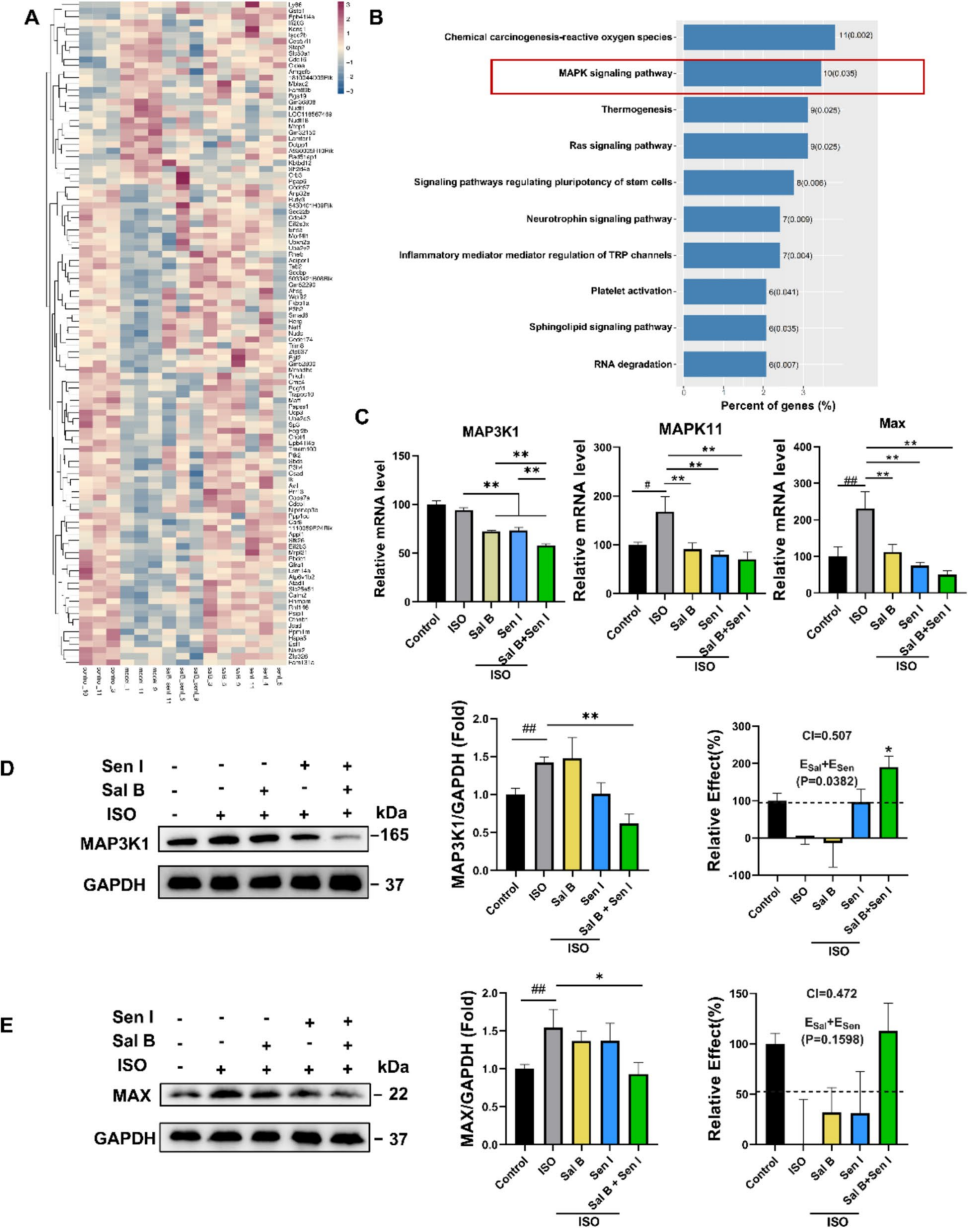
Coadministration of Sal B and Sen I regulates the MAP3K1 signaling. **A** Heat map showing representative differentially expressed genes in cardiac tissue. **B** KEGG pathway analysis of RNA-seq data. **C** Relative levels of MAP3K1, MAPK11 and Max mRNA in mice heart tissue (n = 3 per group). **D**, **E** Representative western blot and quantification analyses of cardiac MAP3K1 (**D**) and MAX (**E**) expression (n = 5 per group). The predicted additive effects for Sal B and Sen I (E_sal_ + E_sen_) is represented by a black dashed line. Sal B, 80 mg/kg/day; Sen I, 50 mg/kg/day; captopril, 20 mg/kg/day. Statistical significance was analyzed using Student’s t-test in (**D,** right and **E**, right) and one-way ANOVA with Tukey’s post hoc test; ^#^P < 0.05, ^##^P < 0.01 when compared with the control group; *P < 0.05, **P < 0.01

Finally, we investigated the potential molecular mechanisms underlying the synergistic anti-hypertrophic effects of Sal B and Sen I. We analyzed the transcriptomic profiles in the heart tissues of ISO-treated mice. A total of 1018 DEGs were identified between ISO-induced mice with and without treatment. Among these, 111 DEGs showed reversed expression between the model group and the Sal B and Sen I co-administration group. From the top-ranked signaling pathways identified via KEGG analysis, we focused on the MAPK signaling pathway, which is crucial in the development and progression of cardiac hypertrophy [41] (Fig. 4A, B). We validated the transcriptional regulation of key components, including MAP3K1, mitogen-activated protein kinase II (MAPK11, also known as p38B), and myc associated factor X (Max), through qPCR (Fig. 4C). We further examined the protein expressions of MAP3K1 and Max in heart tissues. Notably, MAP3K1 protein levels were significantly downregulated in the co-administration group, but not in the single treatment group (Fig. 4D), the similar pattern was also observed for its downstream factor Max (Fig. 4E), indicating that Sal B and Sen I may regulate the expression of MAP3K1 signaling together. Based on transcriptomic findings that highlighted MAPK signaling as a key regulatory axis, we further investigated two critical components of the MAPK network, ERK1/2 and TLR4. The two factors have been reported to function downstream of or in cross-talk with MAP3K1 signaling [42, 43], and both are implicated in the pathogenesis of cardiac hypertrophy [44, 45]. Notably, Sal B significantly inhibited ERK1/2 phosphorylation, whereas Sen I selectively suppressed TLR4 expression (Figure S4). However, the absence of synergistic effects on ERK1/2 and TLR4 by the combination treatment, suggests these two signalings may represent distinct signaling routes for Sal B and Sen I, while MAP3K1 appears to serve as a converging node mediating their cooperative actions in modulating cardiac hypertrophy.

**Fig. 5:**
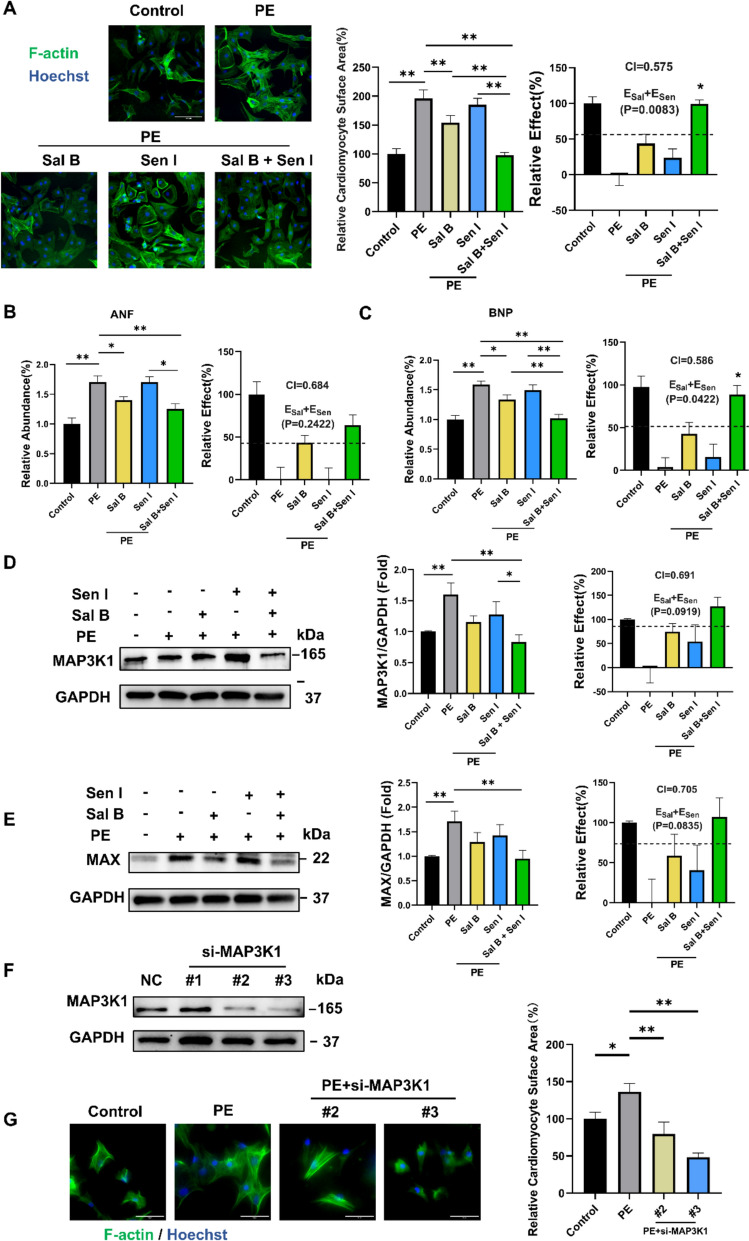
Synergistic effects of Sal B and Sen I in PE-induced hypertrophic NRCMs. **A** Representative fluorescence images and corresponding quantification of phalloidin (F-actin) immunostaining in NRCMs. **B** and **C** Relative levels of ANF and BNP mRNA. **D**, **E** Representative and quantification of the western blot study of MAP3K1 **F** The efficiency of MAP3K1 knockdown in NRCMs. **G** Representative fluorescence images and corresponding quantification of phalloidin (F-actin) immunostaining in NRCMs. (**D**) (n = 5 per group) and MAX (**E**) (n = 6 per group). PE, 100 μM; Sal B, 25 μM; Ros acid, 25 μM. Scale bar, 50 μm. Quantitative data are presented as the mean ± SEM. The predicted additive effects for Sal B and Sen I (E_sal_ + E_sen_) are represented by a black dashed line. Statistical significance was analyzed using Student’s t-test in CI analysis and one-way ANOVA with Tukey’s post hoc test; *P < 0.05, **P < 0.01. Scale bar, 100 μm

Based on the results from the mouse model, we further investigated the synergistic effects of Sal B and Sen I in the PE-induced hypertrophic NRCMs model. Noticeably, the co-administration of Sal B and Sen I showed significantly superior effects in inhibiting the size of cardiomyocytes compared to the individual treatments (Fig. 5A). Furthermore, this combination treatment led to a significant downregulation of hypertrophic markers ANF and BNP (Fig. 5B, C), compared to either compound alone. Additionally, consistent with our findings in the mice model, co-treatment with Sal B and Sen I also inhibited the protein expressions of MAP3K1 and Max (Fig. 5D, E). To further validate the critical role of MAP3K1, we performed siRNA-mediated knockdown experiments in primary NRCMs using three independent siRNA sequences. Among them, siRNA #2 and #3 achieved reduction of MAP3K1 protein levels (Fig. 5F). Importantly, MAP3K1 knockdown significantly attenuated PE-induced cardiomyocyte hypertrophy, as reflected by reduced cell surface area (Fig. 5G). These results provide functional evidence that MAP3K1 is not only associated with, but required for the development of cardiomyocyte hypertrophy, thereby supporting its role as a central mediator of the Sal B and Sen I combination effect. Collectively, these findings reinforce the hypothesis that Sal B and Sen I synergistically attenuate cardiac hypertrophy via coordinated suppression of the MAP3K1 signaling pathway.

### Sal B and Sen I may bind to MAP3K1 together

**Fig. 6:**
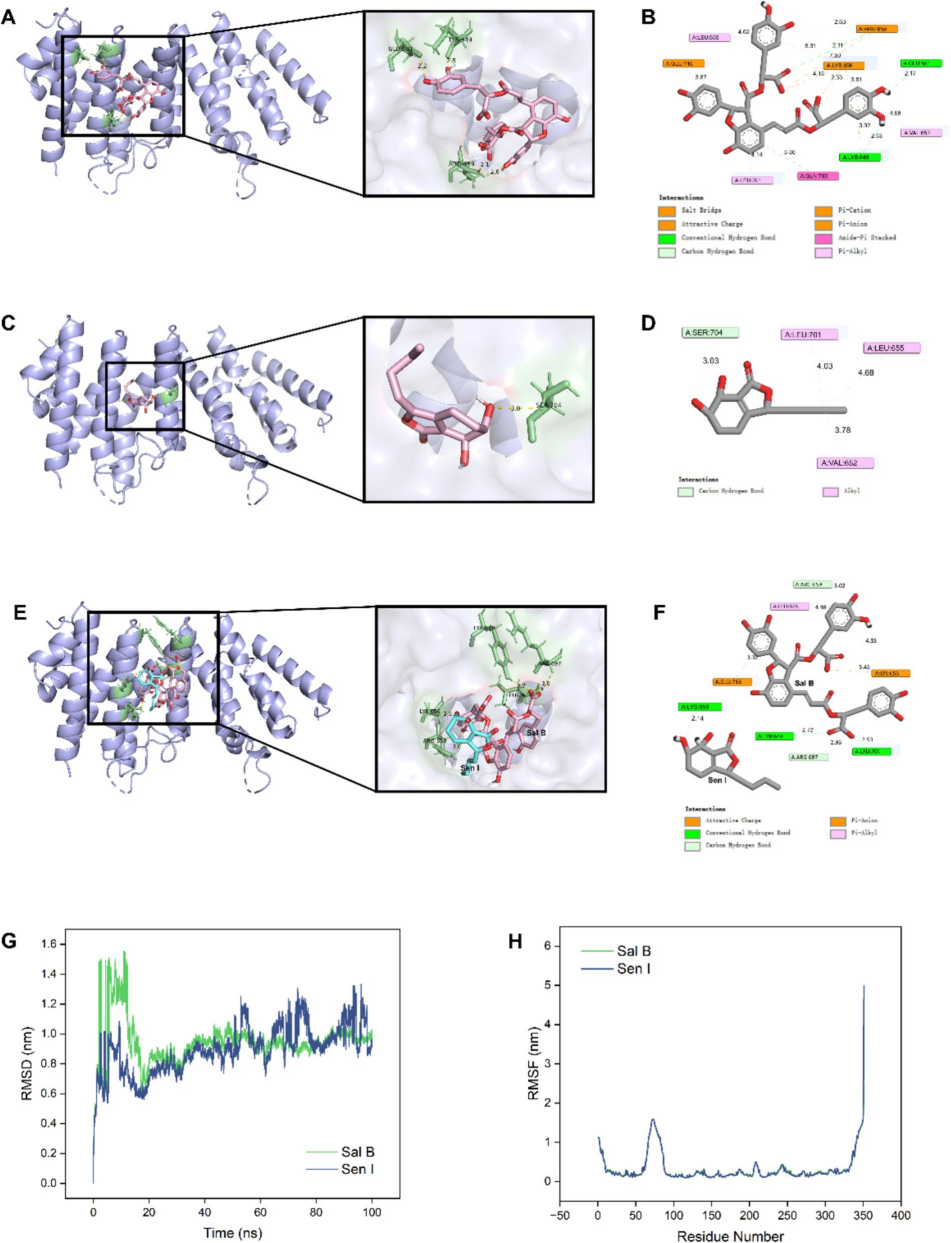
The molecular docking and dynamics simulation results for Sal B and Sen I towards MAP3K1. **A**, **C** and **E** The representative 3D docking modes of MAP3K1 with Sal B (**A**), Sen I (**C**) and both two compounds (**E**), respectively. The green sticks refer to active site residues and dotted yellow lines indicate hydrogen-bonding interactions between protein and ligand. **B**, **D** and **F** The representative 2D protein–ligand interactions for MAP3K with Sal B (**B**), Sen I (**D**) and both two compounds (**F**), respectively. The colored plates refer to active site residues and dashed lines indicate different interaction bonds. **G**, **H** RMSD (**G**) and RMSF (**F**) values of each protein–ligand complex through 100 ns MD simulation

Next, we examined the potential binding between Sal B/Sen I and MAP3K1. As suggested by the molecular docking assay, the predicted binding score for multiple ligands interaction among Sal B, Sen I and MAP3K1 is − 8.103 kcal/mol, which is higher than those of single ligand interactions (Sal B-MAP3K1: − 6.847 kcal/mol; Sen I-MAP3K1: − 5.833 kcal/mol), and provided further insights into their collaborative regulation of MAP3K1 (Table 1). As shown in the representative docking modes of Sal B and Sen I towards MAP3K1, compared to single-ligand docking (Fig. 6A–D), the multiple-ligands docking formed more interactions, including hydrogen-bonding interaction with TYR648, LYS656, ARG697, and LEU701(Fig. 6E, F). It is predicted that Sal B and Sen I can simultaneously bind to MAP3K1, forming a more complex interaction network with additional hydrogen bonds and π-π interactions.

**Table 1:** The docking results of Sal B and Sen I towards MAP3K1

Protein	Docking type	Docking score (kcal/mol)	Ligand	H-bond interactions
MAP3K1	Single ligand	− 6.847	Sal B	GLU581, LYS649, ARG659
		− 5.833	Sen I	SER704
	Multiple ligands	− 8.103	Sal B and Sen I	TYR648, LYS656, ARG659, ARG697, LEU701

Moreover, we performed the molecular dynamics simulation for 100 ns to evaluate the binding stability of Sal B and Sen I with MAP3K1. The plot of RMSD shows that Sal B rapidly decreased after an initial increase, reaching a relatively stable state at around 50 ns, suggesting better binding stability with MAP3K1(Fig. 6G). In comparison, Sen I maintained higher RMSD values with significant fluctuations, indicating unstable binding interactions. The RMSF values for Sal B and Sen I showed similar behavior during the simulation, indicating that the two compounds were combined with similar active sites of MAP3K1 (Fig. 6H). Therefore, the above results suggested that Sal B and Sen I may bind to MAPK3K1 together, and this could explain their synergistic regulation on the expression of MAP3K1 signaling.

## Discussion

As one of most well-established branches of ethnopharmacology globally, TCM offers significant advantages in treating complex diseases which involve multiple biological pathways, such as cardiovascular diseases [46, 47]. However, the intricate chemical composition of naturally derived herbs and materials in TCM often obscures their pharmacological mechanisms [48], leading to ongoing skepticism regarding the efficacy of TCM combinations [49]. In this study, we investigate the effects of GXNT, a botanical drug composed of *Smil* and *Lstr*, on pathological cardiac hypertrophy using both the in vitro and the in vivo models. We also propose a novel synergistic interaction between Sal B and Sen I, two chemical compounds in GXNT, in regulating cardiac hypertrophy.

Pathological cardiac hypertrophy, a hallmark of various cardiovascular diseases, involves maladaptive cardiomyocyte enlargement due to different stressors [50]. Current therapies targeting the renin–angiotensin–aldosterone system, β-adrenergic receptors, or calcium channels often show limited efficacy, benefiting fewer than 50% of patients [3, 51]. In TCM, this condition is perceived as “heart vessel obstruction” (Mai Bi), characterized by blood stasis and qi stagnation, concepts that correlate with modern understanding of microcirculatory dysfunction and neurohormonal imbalance in hypertrophy development. TCM and medicinal herbs offer complementary approaches with notable clinical benefits in China and other countries [52–54]. In classical TCM literatures, *Smil* and *Lstr* are well-known for promoting blood circulation and removing stasis (Huo-Xue Hua-Yu). *Smil* is commonly used for atherosclerosis, myocardial infarction and myocardial ischemia, while *Lstr* benefits ischemic stroke [55–57]. The chemical constituents of both herbs have been thoroughly identified in multiple chemical analyses. *Smil* contains more than 30 lipophilic compounds (including tanshinones, cryptotanshinone, isothanshinones) and over 50 hydrophilic compounds (such as danshensu and salvianolic acids) [58]. Similarly, more than 200 compounds have been isolated from *Lstr*, including phenols, organic acids, phthalides, alkaloids, polysaccharides, ceramides and cerebrosides [59]. Moreover, these two herbs are frequently co-administered in TCM formulas to treat cardiovascular diseases and are believed to provide additional benefits according to TCM combination theory. GXNT, a patented Chinese drug developed based on the *Smil* and *Lstr* formula, has demonstrated clinical efficacies in RCT trials [11, 60]. However, the synergistic pharmacological basis of this combination remains elusive.

To better understand the effects of TCM formulae, it is crucial to identify the active components in the medicinal herbs. However, the complex chemical composition and unclear molecular targets of TCM pose significant challenges to research. In this study, we proposed a strategy of phenotype-based screening in both cell and zebrafish models to identify the active substances in GXNT. Unlike molecular-target based screening, phenotypic screening is better suited for discovering hits with unknown downstream targets. Using F-actin staining, we assessed the cardiomyocyte area to directly evaluate the regulatory effects of GXNT and its major chemical constituents on cardiac hypertrophy. In the zebrafish model, we leveraged the feasibility in live-embryo imaging and measured multiple cardiac function-related parameters in fish lines with fluorescently labeled heart [26, 61]. Interestingly, different active compounds were identified in the two models. This divergence may reflect fundamental differences in both pathological mechanisms and pharmacokinetic properties between the in vitro and in vivo systems.The PE-induced NRCM model predominantly activates metabolic remodeling pathways [62], which may preferentially respond to Sal B treatment. In parallel, the AA-induced zebrafish model develops inflammation-driven cardiac hypertrophy, that appears particularly sensitive to Sen I intervention. These observations align with our subsequent findings showing Sal B’s association with ERK1/2 modulation and Sen I’s connection to TLR4 signaling (Figure S4), though further validation is needed to establish direct causal relationships. Pharmacokinetically, zebrafish’s whole-organism metabolism and tissue distribution may favor Sen I’s bioavailability, while NRCM’s direct cellular exposure may amplify Sal B’s effects. Notably, both compounds demonstrated efficacy in the mouse model, with enhanced protection upon co-administration. These findings underscore the value of multi-model screening for identifying complementary therapeutic mechanisms that collectively address the metabolic and inflammatory processes of cardiac hypertrophy, that reflect the complex disease nature and the advantage of TCM-based interventions.

Both Sal B and Sen I have been reported to exhibit diverse pharmacological activities in previous literatures. Sal B is a water-soluble phenolic acid with suggested benefits for cardiovascular diseases, cancer, aging, and liver fibrosis. Its diverse pharmacological effects are thought to be largely related to its ability to scavenge ROS and inhibit their generation [63, 64]. To further investigate the signaling pathways mediating the synergistic effects of Sal B and Sen I, a transcriptomic sequencing assay was performed, identifying the MAP3K1 pathway as a potential target. MAP3K1 activation promotes downstream kinases MKK4 and MKK7, which activate p38 kinases and JNKs, leading to GATA4-mediated transcription that drives pathological hypertrophy [65]. Previous research showed that the absence of MAP3K1 prevented increases in cardiac mass and myocyte size in a mouse model of hypertrophy induced by the G protein Gαq [7]. The downstream factor Max may also increase ventricular hypertrophy by mediate the transcriptional upregulation of glutaminolysis [66]. Noticeably, a recent study established GXNT’s MEK-ERK1/2 inhibition in cardiac hypertrophy [44], while our study reveals how its compounds Sal B and Sen I synergistically target the upstream factor MAP3K1, providing greater mechanistic resolution of this herbal medicine's polypharmacology. Previous studies revealed that MAP3K1 acts upstream of ERK1/2 to promote ETS2 activation and NFAT-driven transcription, forming a key signaling hub in maladaptive hypertrophy [42]. However, the role of MAP3K1 remains controversial, as heart failure and sudden death was reported in MAP3K1-null mice under pressure overload [10]. Such discrepancies may reflect differential signaling pathways engaged by distinct hypertrophic stimuli, pressure overload (afterload-driven) may activate compensatory or parallel mechanisms not present in neurohormonal models. Additionally, MAP3K1 regulates multiple MAPK branches (ERK, JNK, p38) [67], and its effects may vary depending on target cell type (cardiomyocytes, fibroblasts or endothelial cells), or the degrees of pathway cross-talk. Therefore, the role of MAP3K1 in pathologic cardiac hypertrophy requires further investigation. In our study, co-administration of Sal B and Sen I downregulated both MAP3K1 expression and its downstream factors Max and MAPK11. Importantly, knockdown of MAP3K1 alone effectively attenuated cardiac hypertrophy to a similar extent as the drug combination, suggesting its role as a critical mediator through which Sal B and Sen I exert their therapeutic effects. Molecular docking suggested cooperative binding between Sal B, Sen I and MAP3K1, with enhanced hydrogen bonding implicating allosteric stabilization. Notably, while Sen I exhibited unstable direct binding in simulations, its observed effects may also occur indirectly through suppressing the TLR4/MyD88-MAP3K1 signaling [68]. This parallel regulation may complement Sal B’s more stable direct binding, together producing enhanced inhibition of MAP3K1 and downstream hypertrophic pathways.

Finally, the pharmacological synergy between Sal B and Sen I provides a unique opportunity to bridge TCM principles with modern mechanistic understanding. According to TCM theory, the combination of *Smil* and *Lstr* represents a classical “blood-activating and stasis-resolving” herb pair, historically employed in cardiovascular therapies. Within GXNT, *Smil* is regarded as the “sovereign” (Jun) herb, primarily responsible for the therapeutic action, while *Lstr* serves as the “minister” or “guide” (Chen/Shi) to enhance efficacy and bioavailability. Our findings align with this principle: Sal B, the major compound in *Smil*, significantly suppressed ERK1/2 phosphorylation, implicating its role in oxidative stress and metabolic regulation; Sen I, a key constituent of *Lstr*, downregulated TLR4 expression, consistent with its anti-inflammatory activity. While these two pathways appear to operate independently, their convergence on MAP3K1 signaling highlights a mechanistic synergy that reflects the empirical rationale of TCM compatibility theory.

In conclusion, this study utilized an in vitro NRCM model and an in vivo zebrafish model to systematically assess the effects of the botanical drug GXNT on cardiac hypertrophy, identifying Sal B and Sen I as two primary active compounds through phenotypic screening. Notably, even advanced therapies like angiotensin receptor-neprilysin inhibitors (ARNis), while improving outcomes in heart failure with reduced ejection fraction, exhibit limited efficacy in early-stage hypertrophy and predominantly target neurohormonal pathways [69]. In contrast, the Sal B/Sen I combination identified here addresses exhibit broader modulation of metabolic and inflammatory pathways upstream of hypertrophy, and may serve as potential adjuvants to standard therapies. However, there are limitations in this study. For instance, only compounds with the highest mass spectrometry peaks were selected for screening, potentially overlooking other minor components that may have important pharmacological effects. While lipophilic constituents such as tanshinones from *Smil* are known bioactive compounds [55], the standardized aqueous extraction process used in GXNT preparation would inherently limit their presence in the final formulation. Although our study focused on identifying the major active components in the clinically used GXNT formulation, future phytochemical studies of the raw herbs may provide additional insights. Besides, as we did not conduct quantitative analysis for the amounts of the active compounds in GXNT, a direct comparison between the effects of Sal B and Sen I alone versus the GXNT formulation could not be performed. A more detailed understanding of the pharmacokinetics and pharmacodynamics of Sal B and Sen I in GXNT is warranted for further study. Moreover, the precise mechanism by which Sal B and Sen I regulate MAP3K1 signaling, and how MAP3K1 orchestrates downstream hypetrophic responses still require further elucidation. Despite these limitations, this study provides new perspectives on the pharmacological basis and combination theory of a TCM formulae in treating cardiac hypertrophy.

## Conclusion

GXNT exhibits strong regulatory effects in the treatment of cardiac hypertrophy, with Sal B and Sen I identified as key active compounds. These two components synergistically protect the heart, potentially by modulating the MAP3K1 signaling pathway.

## Supplementary Information

Supplementary Material 1.

## Data Availability

All data involved in this study are available from the corresponding author upon reasonable request.

## Abbreviations

AA: Aristolochic acid A
ANF: Atrial natriuretic factor
Ang II: Angiotensin II
BNP: Brain natriuretic peptide
CI: Combination index
CO: Cardiac output
Dan: Danshensu
DEG: Differentially expressed gene
ED: End-diastole
EPAC: Exchange protein directly activated by cAMP
ES: End-systole
FAC: Fractional area change
FS: Fractional shortening
GXNT: Guanxinning tablet
H&E: Hematoxylin and eosin
hpf: Hours post-fertilization
ISO: Isoproterenol
JNKs: JUN N-terminal kinases
*Lstr*: Ligusticum chuanxiong DC
LV Vol,s: Left ventricular end-systolic volume
LVID,d: Left ventricular internal diameter during diastole
LVMI: Left ventricular mass index
MAP3K1: Mitogen-activated protein kinase kinase kinase 1
MAPK: Mitogen-activated protein kinase
MAPK11: Mitogen-activated protein kinase II
Max: Myc associated factor X
MD: Molecular dynamics
MDA: Malondialdehyde
NFAT: Nuclear factor of activated T cells
NRCMs: Neonatal rat cardiomyocytes
PE: Phenylephrine
RAS: Renin–angiotensin–aldosterone system
RCT: Randomized controlled trials
RMSD: Root-mean-square deviation
RMSF: Root-mean-square fluctuation
ROS: Reactive oxygen species
Ros acid: Rosmarinic acid
Sal B: Salvianolic acid B
SEM: Standard error of the mean
Sen I: Senkyunolide I
*Smil*: *Salvia* *miltiorrhiza* Bunge
SV: Stroke volume
TCM: Traditional Chinese Medicine
