# Supplementary material for: Salvianolic acid B and Senkyunolide I synergistically alleviate cardiac hypertrophy and regulate MAP3K1 signaling
Source: Chin Med. 2025 Sep 28;20:148. doi: 10.1186/s13020-025-01189-9 (PMC12476628; doi:10.1186/s13020-025-01189-9)
Supplement: Supplementary file 1 — Supplementary Material 1. [file 13020_2025_1189_MOESM1_ESM.docx]

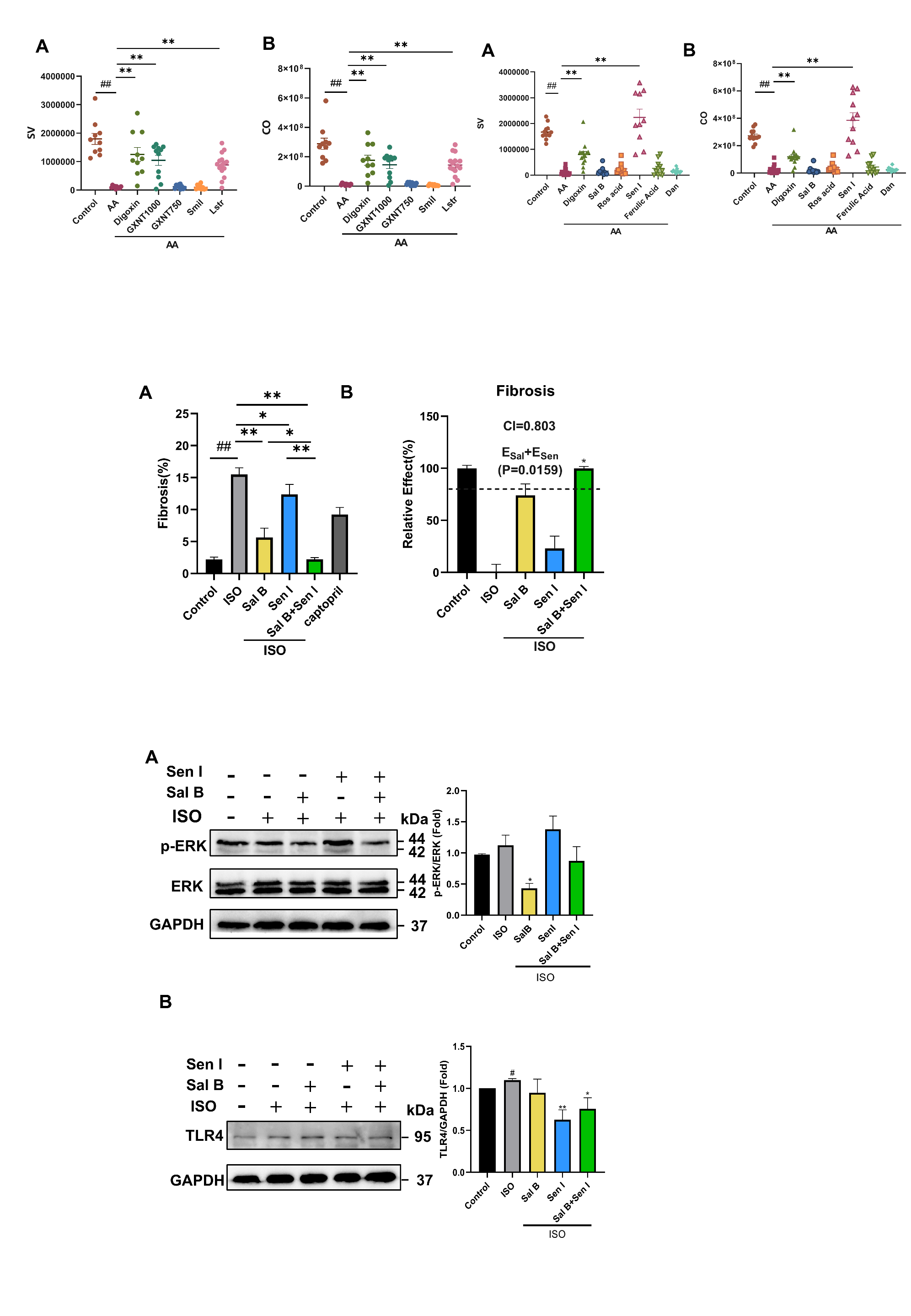


**Figure S1.** Effects of GXNT, *Smil*, and *Lstr* extracts on the zebrafish cardiac hypertrophy model (n =10 per group). (A) SV, stroke volume; (B)CO, cardiac output. AA, 50μM; Digoxin, 10μM; GXNT, 750 or 1000μg/ml ;The concentrations of *Smil* and *Lstr* were 312μg/ml and 463μg/ml respectively. SV, stroke volume; CO, cardiac output. Quantitative data are presented as the mean ± SEM. Statistical significance was analyzed using one-way ANOVA with Tukey’s post hoc test; ##P<0.01 when compared with the control group; **P<0.01 when compared with the AA group.


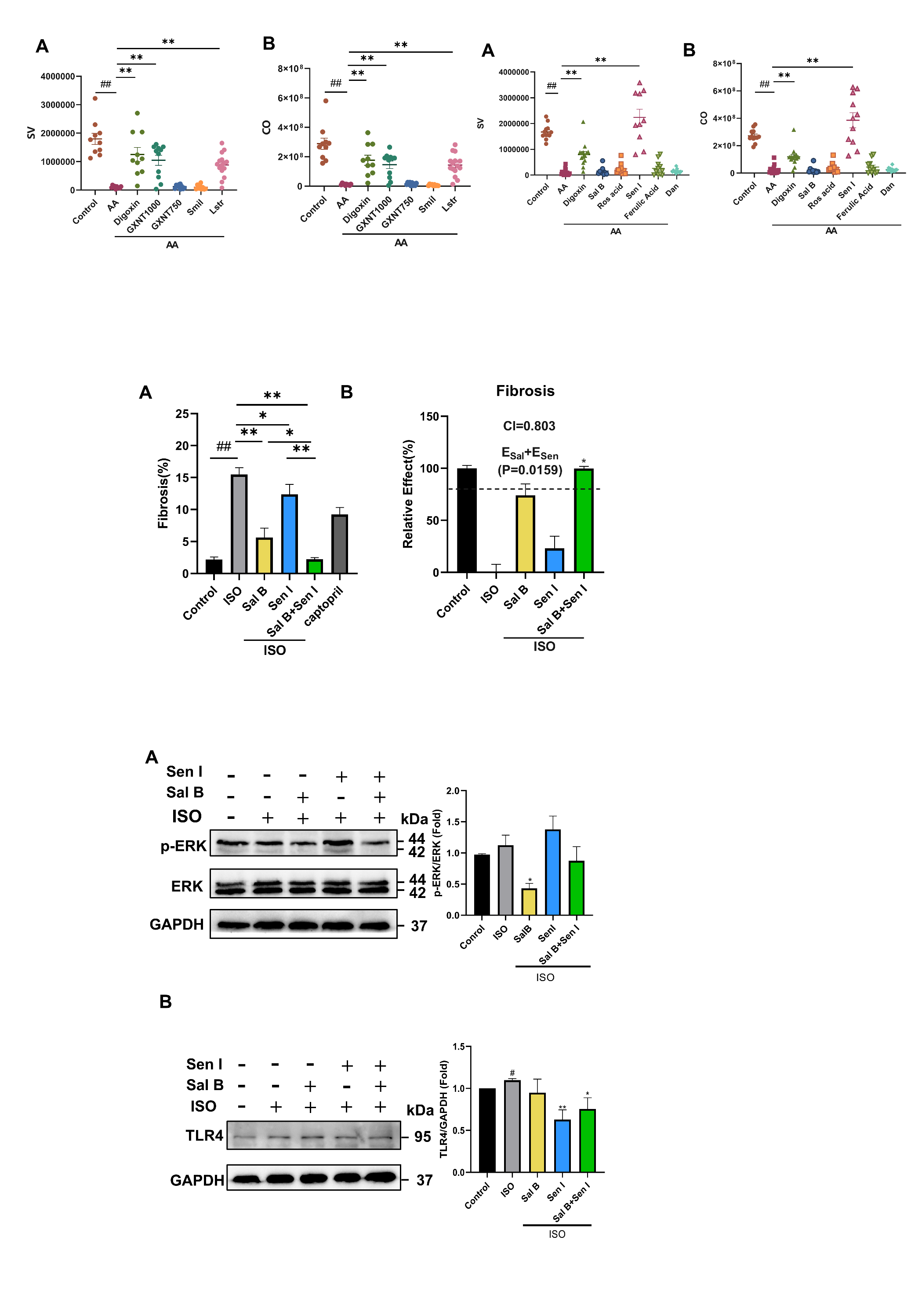


**Figure S2.** Effects of GXNT compounds on the zebrafish cardiac hypertrophy model (n=11-14 per group). (A) SV, stroke volume; (B)CO, cardiac output. The concentration of all compounds is 100μg/ml. Quantitative data are presented as the mean ± SEM. Statistical significance was analyzed using one-way ANOVA with Tukey’s post hoc test; ^#^P<0.05, ^##^P<0.01 when compared with the control group; *P<0.05, **P<0.01 when compared with the AA group; ns, non-significant.


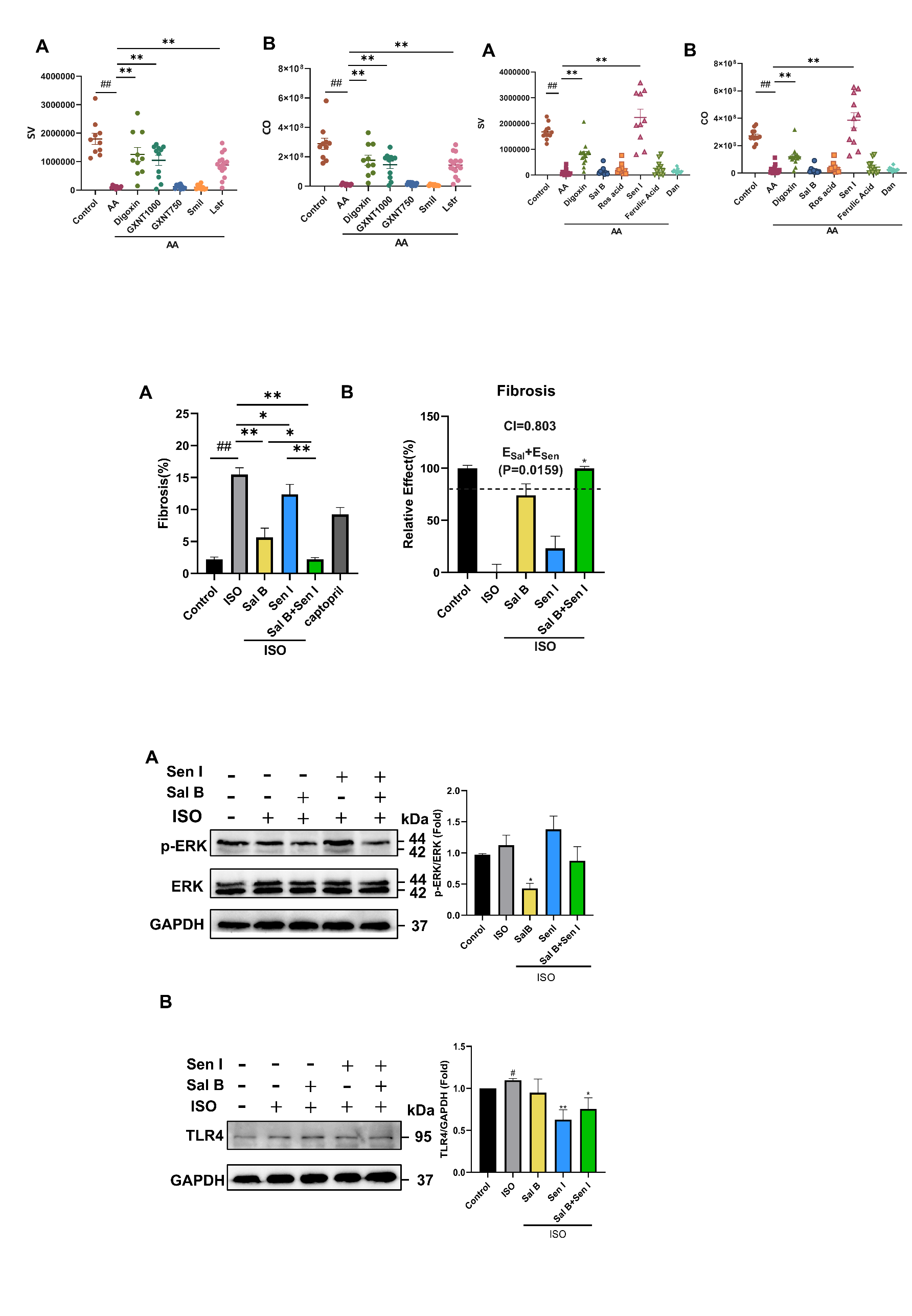


**Figure S3.** Sal B and Sen I cooperatively alleviate cardiac fibrosis in mice. (A) Quantitative analyses of Sirius red staining of heart sections (n=12 per group). (B) Analysis of the interaction between Sal B and Sen I in regulating fibrosis (n=12 per group). The predicted additive effects for Sal B and Sen I (E_sal_+E_sen_) is represented by a black dashed line. Sal B, 80 mg/kg/day; Sen I, 50 mg/kg/day; captopril, 20 mg/kg/day. Statistical significance was analyzed using Student’s t-test in (**B**) and one-way ANOVA with Tukey’s post hoc test; ^##^P<0.01 when compared with the control group; *P<0.05, **P<0.01. Scale bar: 50μm.


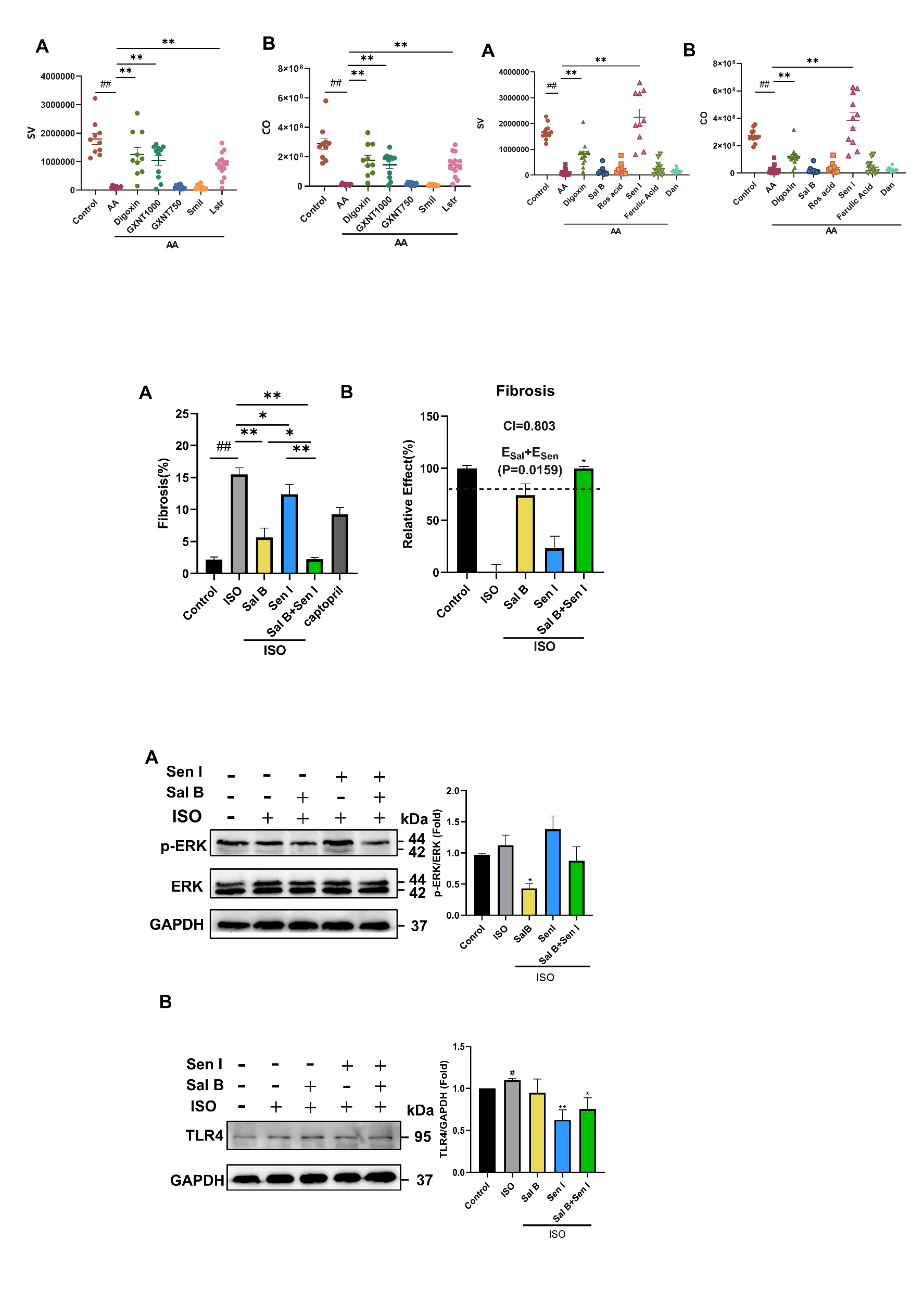


**Figure S4.** Effect of Sal B and Sen I on ERK and TLR4 in mice. (A) Representative western blot of cardiac p-ERK and ERK protein expression (n=3 per group). (B) Representative western blot of cardiac TLR4 protein expression (n=4 per group). Statistical significance was analyzed using one-way ANOVA with Tukey’s post hoc test; ^#^P<0.05, ^##^P<0.01 when compared with the control group; *P<0.05, **P<0.01 when compared with the ISO group.
